# Peanut butter–based formulations of amoxicillin for pediatric applications

**DOI:** 10.1093/inthealth/ihz031

**Published:** 2019-07-31

**Authors:** Helen Tran, Danny Lee, Sarah E Petnic, Julianne A Bozzini, Sangwei Lu

**Affiliations:** Division of Infectious Diseases and Vaccinology, School of Public Health, University of California, Berkeley, CA, USA

**Keywords:** antibiotic, amoxicillin, child-friendly, formulation, pneumonia, peanut butter

## Abstract

**Background:**

Child mortality is a major global health challenge, especially in regions of limited resources. Accessibility to lifesaving medicine and adequate nutrition is essential to reduce child mortality and improve the health and well-being of the world’s most vulnerable children.

**Methods:**

We have developed NutMox, a novel pediatric formulation of the β-lactam antibiotic amoxicillin in a matrix of peanut-based ready-to-use therapeutic food (RUTF) consisting of peanut butter, sugar, vegetable oil, dry milk and vitamins. NutMox is ready to use and thermostable, requires no chewing or pill swallowing and provides both an antibiotic and nutrition.

**Results:**

Amoxicillin in NutMox formulations was stable for at least 12 months at storage temperatures of 4°C, 25°C and 37°C. Amoxicillin formulated in NutMox displayed similar pharmacokinetics in mice to that in suspension.

**Conclusions:**

Our results demonstrated the feasibility of a peanut butter–based matrix for pediatric formulations of amoxicillin, suggesting that such a matrix can serve as a base for delivering medications in addition to its current use as an RUTF.

## Introduction

Despite rapid advances in medicine, child mortality remains a major public health challenge. In 2015, 5.9 million children <5 y of age died worldwide; on average, 16 000 children <5 y of age die each day.^1^ The major infectious causes of mortality are diarrhea and pneumonia, which are estimated to kill >2 million young children each year.^2^ The burden of these diseases is further worsened by malnutrition, as it renders children more vulnerable to diarrhea and pneumonia as well as other infectious diseases. Malnutrition is believed to be a significant contributing factor in close to half of the cases of mortality in children <5 y of age.^3–8^ Therefore, reducing child mortality requires a multidisciplinary approach that simultaneously addresses infection and nutrition. Treating both infection and malnutrition will not only reduce child mortality, but will also improve the lifelong health and well-being of these children.

Much progress has been made in the treatment of childhood infections in recent decades. Pneumonia caused by bacterial pathogens (e.g. *Streptococcus pneumoniae*) can be successfully treated by antibiotics if treatment is delivered in time. The β-lactam antibiotic amoxicillin is recommended by the World Health Organization (WHO) as the first line of treatment for community-acquired pneumonia.^3^ It is also used to treat other common pediatric conditions, such as ear, nose and throat infections. Because of its importance as well as its safety profile, amoxicillin is included in the WHO Model List of Essential Medicines.^9^ Amoxicillin is formulated as a suspension for pediatric applications and has a relatively short shelf life, even with refrigeration. In 2011 a dispersible tablet form of amoxicillin became available, representing a major improvement, as the tablets do not require refrigeration and can be reconstituted in a small amount of clean water or breast milk.^10^

Advances have also occurred in the treatment of severe acute malnutrition in regions of limited resources. A remarkably effective method for treating malnutrition is the peanut butter–based ready-to-use therapeutic food (RUTF) pioneered by Dr. André Briend.^11^ The peanut butter–based products Plumpy’nut (Nutriset, Malaunay, France) and RUTF (Project Peanut Butter, Maplewood, MO, USA) have been proven to be safe and effective and are well received by children in African, Latin American and Asian countries.^12–14^ Furthermore, peanut butter–based RUTFs were more effective at treating malnutrition and reducing mortality when combined with antibiotics.^15^ Recent reports have shown that groups of severely malnourished children receiving an RUTF therapy in conjunction with amoxicillin or cefdinir had a mortality rate of 4.8% and 4.1%, respectively, while the group treated with only the RUTF had a mortality rate of 7.4%.^15,16^ This demonstrates that treating infection and addressing malnutrition work synergistically to improve outcomes for the most vulnerable children.

In this report we aim to describe new therapeutic reagents that treat both infection and malnutrition. To this end, we tested the feasibility of combining amoxicillin with a peanut butter–based RUTF given their importance and efficacy in their current applications. We hypothesized that amoxicillin formulated in a peanut butter–based RUTF would have many desirable features as a pediatric formulation. It should have a favorable taste profile and thermostability and be accessible to young children who are unable to chew or swallow pills. We found that the peanut butter–based amoxicillin formulations, which we named NutMox, were stable at various storage temperatures and demonstrated pharmacokinetics similar to the amoxicillin suspension in a small animal model.

## Materials and methods

### Reagents

Chemicals used in the study were obtained from Sigma-Aldrich (St. Louis, MO, USA) unless stated otherwise. Amoxicillin was obtained from Research Products International (Mount Prospect, IL, USA). Bacterial culture media were from BD Biosciences (Franklin Lakes, NJ). Peanut butter, sugar and vegetable oil were purchased from local grocery stores. The RUTF was generously provided by Project Peanut Butter. Amoxicillin capsules, USP, were purchased from Aurobindo Pharma USA (Dayton, NJ, USA).

### Preparation of amoxicillin formulations

Two peanut butter–based formulations of amoxicillin (NutMox), NutMox-E and NutMox-F, were prepared. NutMox-E consisted of amoxicillin mixed with a peanut butter–based RUTF from Project Peanut Butter (50 mg/g of RUTF). NutMox-F was prepared by mixing amoxicillin with a matrix of peanut butter (37%), sugar (41%) and vegetable oil (22%) to 50 mg/g of matrix. Both formulations were thoroughly mixed to ensure even distribution of amoxicillin in the peanut butter matrices.

### Stability test of NutMox samples

NutMox-E or NutMox-F was aliquoted into Eppendorf tubes, sealed and stored at 4°C, 25°C or 37°C. At 2-month intervals, an aliquot was collected from each sample and stored at −20°C before being used for analysis. As controls, amoxicillin powder and capsules were stored at the same temperatures and sampled according to the same schedule.

### Quantification of amoxicillin in NutMox samples

To quantify amoxicillin levels in NutMox samples, we collected and weighed 0.1–0.15 g of NutMox sample and transferred it to an Eppendorf tube. Milli-Q water that was 10 times the weight of the NutMox sample was added to the Eppendorf tube containing a NutMox sample. The tube was thoroughly mixed via vigorous vortexing for 5 min. A total of 600 μL of thoroughly mixed sample was transferred to a fresh Eppendorf tube and combined with 900 μL of Milli-Q water before again undergoing vigorous vortexing for 5 min. The sample was centrifuged at 13 200 rpm for 5 min and the supernatant was collected for analysis of amoxicillin concentration by a hydroxylamine assay.^17^

The hydroxylamine assay was adapted for a 96-well format based on the method described in the Code of Federal Regulations (USA).^17^ For each NutMox sample, 40 μL of extract prepared as above was added to a well in a 96-well plate. A total of 40 μL of Milli-Q water was then added to each well, followed by 50 μL of neutralized hydroxylamine solution, which was prepared by first neutralizing a 5M hydroxylamine solution to pH 7.0 with 1.27M sodium hydroxide and 0.15M sodium acetate and then diluting with 10 volumes of 19% ethanol. After mixing thoroughly, the plate was incubated at room temperature for 5 min. Following incubation, 50 μL of 0.56M ferric ammonium sulfate in 2.6% sulfuric acid was added to each well and mixed before the plate was incubated at room temperature for 3 min. The optical density at 480 nm (OD_480nm_) was measured in a microplate reader (SpectraMax2, Molecular Devices, Sunnyvale, CA, USA). On each plate, serial dilutions of an amoxicillin standard solution were included to construct a standard curve. The amoxicillin concentration of each sample was determined by comparing the OD_480nm_ of the sample to the standard curve.

The amoxicillin in NutMox samples was quantified by ultra-performance liquid chromatography (UPLC) coupled with ultraviolet detection (UPLC/UV). To prepare NutMox samples for analysis by the UPLC/UV method, we weighed 0.1 g of NutMox sample and transferred it to a 50 mL centrifuge tube with 10 mL of hexane. The mix was vortexed for 3 min before adding 10 mL of water and shaking for 30 min to extract amoxicillin. The samples were centrifuged at 10 000 rpm and the top hexane layer was removed. A 500 μL aliquot of the bottom water layer was filtered through a 0.22 μM Ultrafree centrifugal filter tube (EMD Millipore, Billerica, MA, USA) at 10 000 rpm for 3 min. The filtrate was transferred to a UPLC sample plate for UPLC analysis.

Extracted amoxicillin was loaded onto Luna 3 μm C18(2), 100 A, 100×4.6 mm columns (Phenomenex, Torrance, CA, USA) on an Acquity UPLC (Waters, Milford, MA, USA) at 40°C. Mobile phase A consisted of 0.4% formic acid in water (pH 3.2) and mobile phase B consisted of methanol:acetonitrile (75:25 v/v). The gradient was run at 0.7 mL/min for 2 min from 100% A, 0% B to 85% A, 15% B; 0.7 mL/min for 4 min from 85% A, 15% B to 80% A, 20% B; 0.7 mL/min for 0.1 min from 80% A, 20% B to 5% A, 95% B; 1.0 mL/min for 2.9 min at 5% A, 95% B; 1.0 mL/min for 0.1 min from 5% A, 95% B to 100% A; 1.0 mL/min for 3.9 min at 100% A and 0.7 mL/min for 1 min at 100% A. The UV spectrum was collected on a Waters photodiode array detector using the Waters Empower 2 data system. The amoxicillin levels were determined by comparing to a standard curve constructed with the corresponding peanut butter matrix spiked with amoxicillin.

### Pharmacokinetics of amoxicillin in mice

The pharmacokinetics of amoxicillin was analyzed in mice. All procedures involving mice were approved by the Animal Use and Care Committee of the University of California, Berkeley. CD-1 mice, 8–10 weeks old, were obtained from Charles River (Kingston, NY, USA). Mice were fasted 12–16 h before administration of amoxicillin formulations. Water was given ad libitum. Amoxicillin in various formulations (NutMox-E, NutMox-F or saline suspension) was given to mice intragastrically via a feeding needle to a dosage of 30 mg/kg of body weight. At 1, 3 or 5 h after feeding, blood was collected from groups of 10 mice/formulation and heparin was added to prevent coagulation. Blood was spun at 8000 rpm for 5 min, then the plasma was transferred to a fresh tube and stored at –20°C.

The level of amoxicillin in the mouse plasma was measured by liquid chromatography coupled with tandem mass spectrometry (LC-MS/MS). A total of 50 μL of plasma sample were combined with 50 μL of water and 50 μL of cephalexin in methanol (MeOH) and water at a 1:1 ratio was used as the internal control. For each sample, 0.6 mL of MeOH was added before the samples were vortexed for 3 min. After centrifugation at 10 000 rpm for 5 min at 4°C, 500 μL of supernatant was transferred to a fresh tube and dried at 35°C to dryness. The samples were reconstituted with 200 μL of MeOH and water at a 1:1 ratio with 0.5% formic acid and vortexed for 2 min to resuspend the samples.

High-performance liquid chromatography (HPLC) was performed on a system consisting of an SCL-10A controller, LC-10A pump and SIL-10A autosampler (all from Shimadzu, Kyoto, Japan). A Gemini NX-C18, 50×4.6 mm, 5 μm column (Phenomenex) was used for separation at the ambient temperature with 1–5 μL of each sample. The HPLC was run using 0.1% formic acid in water as phase A and MeOH as phase B at the following gradients: 1.2 min at 86% A, 14% B; 1.3 min from 86% A, 14% B to 25% A, 75% B; 0.3 min at 25% A, 75% B; 0.1 min from 25% A, 75% B to 0% A, 100% B; 0.3 min at 0% A, 100% B; 0.1 min from 0% A, 100% B to 95% A, 5% B and 1.5 min at 95% A, 5% B. Mass spectrometry was carried out using an API 4000 LC-MS/MS System (SCIEX, Framingham, MA, USA) in electrospray ionization positive ion detection mode at 500°C. The amoxicillin level was determined using peaks at m/z 366.1–208.0, with m/z 348.1–174.1 from cephalexin as the internal control.

## Results

### Design and formulation of peanut butter–based formulations of amoxicillin

The goal of the project was to develop a novel formulation of amoxicillin that supplied both amoxicillin and nutrition. Ideally it would be ready to use without further preparation, stable without refrigeration and able to be taken by young children without chewing. In this study we tested two peanut butter–based matrices as the base for a child-friendly formulation of amoxicillin, which we named NutMox. To our knowledge, until now, peanut butter has not been used to formulate pharmaceuticals and its suitability as a matrix for antibiotics has not been tested. We reasoned that the low water and high fat content of a peanut butter matrix would likely be beneficial to the stability of the pharmaceuticals it carried.

We prepared two formulations of NutMox: NutMox-E and NutMox-F. NutMox-E used RUTF from Project Peanut Butter, a nonprofit organization treating malnutrition in Malawi, Sierra Leone and Ghana.^18^ NutMox-F was formulated in the laboratory using peanut butter, sugar and vegetable oil without dry milk or vitamins. We decided to test both formulations because NutMox-E would include the full RUTF, while NutMox-F would be easier to prepare and less expensive to produce. We tested each formulation at 50 mg amoxicillin per gram of matrix.

### Quantifying amoxicillin level in peanut butter matrix

Peanut butter is a high-fat substance, and the amoxicillin in NutMox needed to be extracted from peanut butter before being measured. Amoxicillin is soluble in water to approximately 3 mg/mL. Based on this known solubility, we developed an aqueous extraction procedure that extracted amoxicillin from peanut butter matrices through a series of water dilutions and extractions (see Materials and methods). Through dilutions, the amoxicillin concentration was reduced from the initial 50 mg/g of matrix to 2 mg/ml in water. We found that the procedure could effectively recover amoxicillin from the peanut butter matrices with a recovery rate of >90% (data not shown). Extracted samples were then analyzed by a colorimetric assay using hydroxylamine and ferric ammonium sulfate.^17^ The reaction has an excellent linear range around the concentrations of 1–2 mg/ml of amoxicillin found in our samples.

Samples used for UPLC/UV analysis were prepared using a modified extraction method to recover amoxicillin into aqueous solution free of contaminating fat and proteins. First, NutMox samples were extracted with water to recover amoxicillin. The aqueous extracts were then treated with hexane to remove the fatty contents. Subsequently aqueous extracts were filtered before being used for UPLC analysis. The recovery rate for the method was >55% (data not shown).

### Stability of amoxicillin in the peanut butter matrix

**Figure 1.**
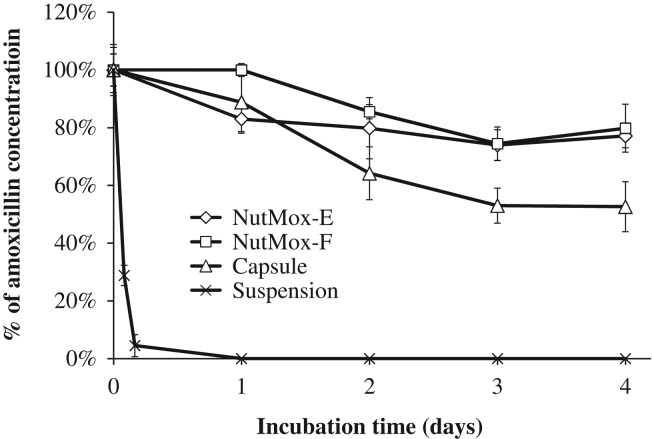
Stability of amoxicillin at 90°C in NutMox-E, NutMox-F, capsule or saline suspension. NutMox-E, NutMox-F, capsules and amoxicillin suspension were incubated at 90°C, and aliquots were sampled after various time periods. The amoxicillin level was determined in each sample. The level of amoxicillin after incubation is shown as a percentage of the initial level. Results are the average of experiments and error bars represent standard deviations.

Stability of the amoxicillin in the NutMox formulations was a key requirement for its preclinical development. A standard stability test is typically conducted by storing the pharmaceutical for a period of several months to a year. Due to the extended duration of the test, we first tested the stability of amoxicillin at elevated temperatures to assess the effect of the peanut butter matrix on amoxicillin. NutMox-E and NutMox-F were subjected to 90°C and compared with amoxicillin capsules and suspension. Aliquots were collected from each sample at various time points and amoxicillin levels were determined by the hydroxylamine assay (Figure 1). Of the four formulations tested, the amoxicillin level in the aqueous suspension became undetectable after 1 h of exposure. In contrast, amoxicillin in capsules and in peanut butter matrices survived the heat treatment to various extents. After 4 d, the amoxicillin level in the NutMox-E and NutMox-F was approximately 80% of the initial level, while the amoxicillin level in the capsules was approximately 50% of the initial level (Figure 1).

Given the stability of amoxicillin in NutMox at 90°C, we initiated a 12-month stability test of NutMox-E, NutMox-F and amoxicillin capsules. NutMox-E, NutMox-F and capsules were stored in airtight containers at 4°C, 25°C and 37°C. Aliquots were collected from each sample bimonthly and processed for quantification of the amoxicillin level by UPLC/UV and the amoxicillin level in each sample was listed as a percentage of the prestorage level (Table 1). As shown in Table 1, amoxicillin was stable in all matrices and temperatures over the 12-month period.

**Table 1.** Stability of formulations of amoxicillin at different temperatures. Relative amoxicillin levels in each formulation are compared to 0-month samples and shown as averages (in %) with a 90% confidence interval. The experiment was performed three times and these results are from a representative experiment

Formulation	4°C	25°C	37°C
Capsule			
0 month	100.0 (95.5 to 104.5)	100.0 (95.3 to 104.7)	100.0 (95.8 to 104.2)
2 months	112.8 (112.5 to 113.0)	109.0 (108.8 to 109.3)	104.1 (103.7 to 104.5)
4 months	105.0 (104.7 to 105.3)	104.4 (104.1 to 104.7)	102.3 (102.0 to 102.5)
6 months	113.8 (113.5 to 114.0)	119.3 (119.0 to 119.6)	105.4 (105.0 to 105.7)
8 months	108.6 (108.1 to 109.1)	109.4 (109.2, 109.6)	106.3 (106.1 to 106.5)
10 months	115.9 (115.5 to 116.2)	113.1 (112.9 to 113.3)	101.3 (100.9 to 101.6)
12 months	115.8 (115.3 to 116.3)	113.8 (113.6 to 114.0)	97.4 (97.0 to 97.7)
NutMox-E			
0 month	100.0 (100.2 to 99.8)	100.0 (100.2 to 99.8)	100.0 (100.2 to 99.8)
2 months	101.1 (102.6 to 99.7)	101.6 (101.8 to 101.4)	106.8 (111.5 to 102.2)
4 months	102.5 (104.0 to 101.0)	104.7 (112.1 to 97.3)	105.1 (106.8 to 103.5)
6 months	100.8 (101.5 to 100.1)	103.1 (105.0 to 101.1)	104.0 (104.4 to 103.6)
8 months	101.7 (104.9 to 98.5)	103.9 (104.7 to 103.1)	109.2 (110.2 to 108.3)
10 months	103.5 (104.8 to 102.2)	98.4 (107.6 to 89.2)	106.3 (108.2 to 104.3)
12 months	105.0 (105.8 to 104.2)	101.4 (106.0 to 96.7)	104.6 (106.0 to 103.1)
NutMox-F			
0 month	100.0 (100.4 to 99.6)	100.0 (100.4 to 99.6)	100.0 (100.4 to 99.6)
2 months	103.3 (105.5 to 101.1)	101.5 (103.7 to 99.3)	101.6 (102.5 to 100.8)
4 months	105.2 (106.2 to 104.3)	102.1 (105.3 to 98.8)	101.4 (104.1 to 98.8)
6 months	104.3 (108.1 to 100.4)	102.5 (106.1 to 98.9)	100.1 (100.7 to 99.6)
8 months	103.6 (104.2 to 103.0)	103.9 (104.3 to 103.6)	99.1 (99.8 to 98.4)
10 months	101.5 (104.6 to 98.4)	101.6 (103.4 to 99.9)	98.8 (99.1 to 98.5)
12 months	100.1 (101.8 to 98.4)	102.3 (106.5 to 98.1)	98.5 (98.9 to 98.0)

### Pharmacokinetics of amoxicillin in mice

**Figure 2.**
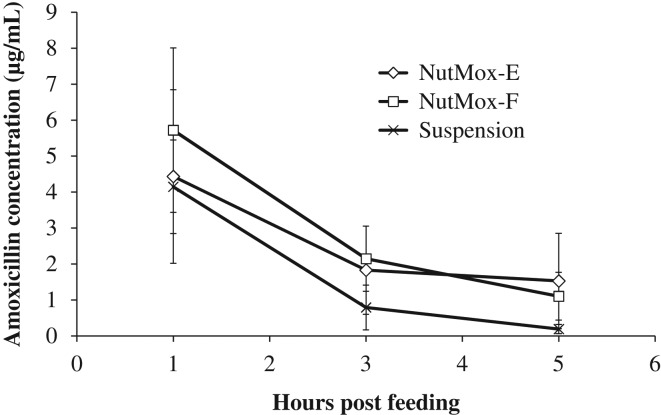
Pharmacokinetics of amoxicillin in mice given amoxicillin in NutMox-E, NutMox-F or saline. Blood was collected 1, 3 and 5 h after feeding. Plasma amoxicillin levels were measured by LC-MS. The experiment was performed three times and these results are from a representative experiment.

As new formulations of amoxicillin, NutMox-E and NutMox-F needed to demonstrate pharmacokinetics equivalent to the currently approved formulations. We tested NutMox-E and NutMox-F in mice and compared them to an amoxicillin suspension, the most common form of pediatric formulation in many countries. NutMox-E and NutMox-F were prepared as described (see Materials and methods). All formulations of amoxicillin were administrated orally at 30 mg/kg body weight, the dosage recommended for pediatric patients. Feeding needles were used to administer each formulation to ensure that a full dose was given and that the time of administration could be accurately recorded. At 1, 3 and 5 h after the administration of amoxicillin formulations, blood was collected and used for measuring plasma amoxicillin levels by LS-MS/MS. The pharmacokinetics of all amoxicillin formulations were similar (Figure 2). The amoxicillin levels were the highest at 1 h, the first time point sampled, and declined rapidly over the next 4 h. By 5 h postadministration, the amoxicillin level was slightly above or near the level of detection. The amoxicillin levels from the NutMox formulations were slightly higher than those of the suspension at 3 and 5 h postadministration and the difference was statistically significant (p<0.05, Student’s t-test).

## Discussion

We report here a study developing new therapeutic reagents to reduce child mortality by combining treatments for infection and malnutrition. Since the target patient populations were at-risk children in resource-limited regions, there were several considerations in the design of the project to address the serious challenges faced by the target patients. First, the new formulations had to be stable and ready to use since refrigeration and clean water suitable for pharmacy use are not always available in resource-poor regions. Second, the formulations needed to be child-friendly and able to be taken by young children who cannot yet chew or swallow bills. Third, the formulations needed to be inexpensive and easy to manufacture. We explored the amoxicillin and peanut butter–based RUTF combination as a proof of concept since both have been widely used in pediatric populations. Here we have shown that it is feasible to use peanut butter as a matrix to formulate amoxicillin. The resulting formulations demonstrated excellent stability during storage and similar pharmacokinetics to the amoxicillin suspension in a small animal model.

Two peanut butter–based amoxicillin formulations, NutMox-E and NutMox-F, were tested in this study. Both contained peanut butter, sugar and vegetable oil, and NutMox-E contained additional dry milk powder and vitamins. In this initial study, we formulated the amoxicillin at 50 mg/g of matrix, which contained a dosage of 250 mg of amoxicillin in 5 g of matrix. This would allow a child to receive a complete dose with relative ease; however, the ratio of amoxicillin or other active pharmaceutical ingredients (APIs) can be easily lowered to provide more nutrition in each dose of medication.

Both NutMox-E and NutMox-F demonstrated excellent stability at 4°C, 25°C and 37°C for 12 months (Table 1). The relative amoxicillin level was within the range required by the U.S. Food and Drug Administration (80–125%, 90% confidence interval).^19,20^ The stability of both formulations was comparable to that of capsules, a currently approved formulation of amoxicillin.

In addition to keeping the APIs stable, it is essential for a new formulation of an approved active ingredient to demonstrate bioequivalence to the existing formulation. Analyses were carried out in mice to compare the pharmacokinetics of amoxicillin from NutMox-E and NutMox-F to that of a saline suspension. Amoxicillin delivered from all formulations reached a peak plasma concentration rapidly and at the first sampling time point of 1 h (Figure 2). The peak plasma level of amoxicillin was similar for all formulations, with no statistically significant difference between formulations (Figure 2). This indicates that the peanut butter matrix did not interfere with the absorption of amoxicillin despite its viscosity and high fat content. The plasma level of amoxicillin declined in all formulations at subsequent time points; however, the decline in amoxicillin level from NutMox-E and NutMox-F was slower than that from the saline suspension (Figure 2). This slower decline resulted in an increased area under the curve (AUC) for the NutMox formulations (112% and 142% for NutMox-E and NutMox-F, respectively. It is possible that NutMox is retained in the gastrointestinal tract longer than the saline suspension and thus is absorbed more. While better retention of the NutMox formulations can be considered an advantage, the dosage of amoxicillin may need to be adjusted to achieve bioequivalence with the amoxicillin suspension. NutMox-E and NutMox-F were not compared with amoxicillin capsules in mice because the small body size of mice does not allow capsules formulated for humans to be tested. NutMox-E and NutMox-F will be compared to capsules or tablets when the study progresses to human studies.

Although peanut butter was initially selected as the base for amoxicillin formulations because of its use in RUTFs and its acceptability by children, we have found that it also functions as a thermoprotectant. When exposed to an extreme temperature of 90°C, the relative level of amoxicillin in the NutMox formulations was approximately 80% after 4 d of heat exposure, whereas the relative level of amoxicillin in capsules dropped to approximately 50% (Figure 1). Although drugs are not expected to be exposed to 90°C temperatures, the results indicate that peanut butter may function as a thermoprotectant, a very desirable property for a drug excipient. The protective effect is most likely due to its high fat content and low moisture level. This is consistent with previous findings that microorganisms are more heat resistant in peanut butter than in aqueous solutions. The thermoprotective property of peanut butter suggests that it can be used for formulating other drugs and may render temperature-sensitive APIs more stable at elevated temperatures.

The effectiveness of oral medications depends on absorption. The APIs of medications need to be retained by the gastrointestinal tract and absorbed into the bloodstream before they can be effective. Previous studies have shown that the intestinal linings of severely malnourished children are damaged, making it difficult for the children to absorb medications.^6,7,21,22^ We have found that peanut butter matrix helps amoxicillin to be retained longer in mice, and this property may help malnourished children better absorb amoxicillin. In addition, it may be possible to further improve gut health and drug absorption by supplementing NutMox formulations with probiotics, as peanut butter is a protective matrix for bacteria and supplemented beneficial bacteria may be able to survive without refrigeration.

Peanuts are widely consumed globally. According to the most recent data available from the U.S. Department of Agriculture, peanut production was 44.86 million metric tons worldwide in 2016–2017^23^ and 2.36 million metric tons in the USA in 2014.^24^ In spite of being a common food, peanuts can cause allergic reactions and trigger anaphylactic attacks in severe cases. However, allergies should not prevent the use of peanuts in pharmaceutical applications. The incidence of peanut allergy is estimated to be 0.4–5% in the USA, depending on methodology, survey population and time period.^25^ The incidence of peanut allergy is much lower in developing countries and peanut butter–based RUTFs have been successfully used in millions of children for several decades. Furthermore, new evidence suggests that an avoidance strategy may not be the most effective way to prevent severe allergy. In the clinical trial Learning Early about Peanut Allergy (LEAP), involving 530 infants, the prevalence of peanut allergy at 60 months of age was 13.7% in the avoidance group and 1.9% in the consumption group (p<0.001).^26^ In 2017, a National Institute of Allergy and Infectious Diseases–sponsored expert panel released a recommendation to introduce peanuts early in infants with an elevated risk for peanut allergy.^27^ This change in strategy can help reduce the incidence of peanut allergy and increase acceptance for peanut butter–based pediatric formulations.

We believe that the novel peanut butter–based formulations we are developing to address infection, malnutrition and gut health may provide a flexible platform for drug formulations and be a useful tool in treating severe malnutrition and reducing child mortality in the most vulnerable populations of the world. Many challenges remain before NutMox becomes a viable option for pediatric use in resource-limited regions. For example, it is important that patients complete a treatment course for any antibiotic to prevent the development of antibiotic resistance. To help ensure patient compliance, we have designed a packaging system for NutMox consisting of clearly labeled packets for two doses per day for a 5-d treatment course.^28^ The packaging allows the caregivers to easily track if a dose is taken and if the treatment course is complete.^28^ Another challenge is the quality and cost of peanut butter. Peanut butter used for formulating NutMox needs to be free of aflatoxin and contaminating bacteria. Procedures established for the manufacturing and quality control of peanut butter–based RUTFs will be used as references. It is not yet known how the cost of NutMox will compare with amoxicillin tablets or suspension since the cost is determined by many factors, including the cost of APIs and excipients, the manufacturing process, market size, distribution network and other factors determined by end-users’ locations and demographics. Despite these challenges, we believe a stable and ready-to-use pediatric formulation of amoxicillin should help make amoxicillin more accessible to children who need this lifesaving medicine.

## Conclusions

In this study we used amoxicillin and peanut butter–based RUTFs as a proof of concept for formulating child-friendly therapeutic reagents that treat diseases and provide nutrition in order to reduce child mortality. The same concept can be used to develop other combinations to treat diseases that are devastating to young children in resource-limited regions. For example, it may be possible to combine APIs that treat human immunodeficiency virus, tuberculosis and malaria with therapeutic food, including peanut butter–based RUTFs. We have shown here that the stability and pharmacokinetic data support such formulations. The next key steps include navigating the regulatory approval processes of the countries and regions where the new formulations will be used and working with local and international manufacturers to design manufacturing processes that satisfy the regulatory requirements.

**Authors’ contributions:** SL conceived the study. SL and HT designed the study protocol. HT, DL, SEP and JAB carried out the stability testing of amoxicillin. HT, SEP and SL carried out the pharmacokinetics analysis in mice. SL drafted the manuscript. All authors read and approved the final manuscript.

**Acknowledgements:** The authors would like to thank Drs. Lee Riley and Veronica Miller of the University of California, Berkeley and Forum for Collaborative Research for their insightful advice and discussions. We are also grateful to Dr. Mark Manary of the Washington University School of Medicine for his suggestions and for providing the peanut butter RUTF samples of Project Peanut Butter for use in this study. The analysis of amoxicillin by UPLC/UV and mass spectrometry was provided by XenoBiotic Laboratories (Plainsboro, NJ, USA).

**Funding:** The study was supported by the Bill and Melinda Gates Foundation (grant OPP1128882).

**Competing interests:** None declared.

**Ethical approval:** All animal studies were approved by the University of California, Berkeley Animal Care and Use Committee and performed following the guidelines of the Office for Animal Care and Use.
